# Alcohol and mortality in Russia: prospective observational study of 151 000 adults

**DOI:** 10.1016/S0140-6736(13)62247-3

**Published:** 2014-04-26

**Authors:** David Zaridze, Sarah Lewington, Alexander Boroda, Ghislaine Scélo, Rostislav Karpov, Alexander Lazarev, Irina Konobeevskaya, Vladimir Igitov, Tatiyana Terechova, Paolo Boffetta, Paul Sherliker, Xiangling Kong, Gary Whitlock, Jillian Boreham, Paul Brennan, Richard Peto

**Affiliations:** a Russian Cancer Research Centre, Moscow, Russia; b Clinical Trial Service Unit and Epidemiological Studies Unit (CTSU), Nuffield Department of Population Health, Oxford University, Oxford, UK; c International Agency for Research on Cancer, Lyon, France; d Institute of Cardiology, Tomsk Research Centre, Tomsk, Russia; e Altai Branch of the Russian Cancer Research Centre, Barnaul, Russia; f Ichan School of Medicine at Mount Sinai, New York, NY, USA

## Abstract

**Background:**

Russian adults have extraordinarily high rates of premature death. Retrospective enquiries to the families of about 50 000 deceased Russians had found excess vodka use among those dying from external causes (accident, suicide, violence) and eight particular disease groupings. We now seek prospective evidence of these associations.

**Methods:**

In three Russian cities (Barnaul, Byisk, and Tomsk), we interviewed 200 000 adults during 1999–2008 (with 12 000 re-interviewed some years later) and followed them until 2010 for cause-specific mortality. In 151 000 with no previous disease and some follow-up at ages 35–74 years, Poisson regression (adjusted for age at risk, amount smoked, education, and city) was used to calculate the relative risks associating vodka consumption with mortality. We have combined these relative risks with age-specific death rates to get 20-year absolute risks.

**Findings:**

Among 57 361 male smokers with no previous disease, the estimated 20-year risks of death at ages 35–54 years were 16% (95% CI 15–17) for those who reported consuming less than a bottle of vodka per week at baseline, 20% (18–22) for those consuming 1–2·9 bottles per week, and 35% (31–39) for those consuming three or more bottles per week; trend p<0·0001. The corresponding risks of death at ages 55–74 years were 50% (48–52) for those who reported consuming less than a bottle of vodka per week at baseline, 54% (51–57) for those consuming 1–2·9 bottles per week, and 64% (59–69) for those consuming three or more bottles per week; trend p<0·0001. In both age ranges most of the excess mortality in heavier drinkers was from external causes or the eight disease groupings strongly associated with alcohol in the retrospective enquiries. Self-reported drinking fluctuated; of the men who reported drinking three or more bottles of vodka per week who were reinterviewed a few years later, about half (185 of 321) then reported drinking less than one bottle per week. Such fluctuations must have substantially attenuated the apparent hazards of heavy drinking in this study, yet self-reported vodka use at baseline still strongly predicted risk. Among male non-smokers and among females, self-reported heavy drinking was uncommon, but seemed to involve similar absolute excess risks.

**Interpretation:**

This large prospective study strongly reinforces other evidence that vodka is a major cause of the high risk of premature death in Russian adults.

**Funding:**

UK Medical Research Council, British Heart Foundation, Cancer Research UK, European Union, WHO International Agency for Research on Cancer.

## Introduction

By west European standards, Russian adults, particularly men, have a very high risk of premature death, which has fluctuated sharply in recent decades. At 2005 mortality rates, for example, only 7% of UK men but 37% of Russian men would die before the age of 55 years (appendix pp 3–5).^1^ Strong alcoholic drink, mainly vodka, is a major cause of the high risk of premature death in Russian adults.^2^, ^3^, ^4^, ^5^, ^6^, ^7^, ^8^, ^9^, ^10^, ^11^ Since 2005, Russian consumption of spirits and male mortality before age 55 years both decreased by about a third (appendix pp 5–6), but are still substantial. The effects of alcohol on mortality need to be assessed both by large retrospective studies of people who have already died (in which information on previous consumption is sought retrospectively from informants who knew the dead person) and by large prospective studies. Both retrospective and, particularly, prospective studies are liable to under-estimate the individual risks and the alcohol-attributable population risks, but prospective studies avoid some of the potential biases of retrospective studies.

A retrospective study of 50 000 deaths in three typical Russian cities (Barnaul, Byisk, and Tomsk) sought information from surviving family members about the drinking habits of the dead person.^10^ Such reports may well understate actual consumption, but even in those who had died of diseases that were deemed unlikely to be much related to alcohol use, 47% of the men and 11% of the women were reported to have drunk at least a bottle of vodka a week, and many were reported by their families to have drunk on average about a bottle of vodka a day. The proportions reported to have done so were, however, substantially larger than this in those who had died from external causes (accident, poisoning, suicide, or homicide) or eight particular disease groupings (cancer of the upper aerodigestive tract or liver, other liver disease, tuberculosis, pneumonia, acute pancreatitis, acute ischaemic heart disease other than myocardial infarction [ICD-10 I24^12^], and ill-specified conditions, appendix p 10), showing that mortality from these causes was substantially increased in drinkers.

Both retrospective and prospective studies are limited by the unreliability of the information they obtain about alcohol use and by the variability of drinking patterns, but have complementary strengths. Retrospective studies can obtain information on large numbers of deaths relatively quickly, and approach the ideal of sampling all deaths (although informants may be unavailable, or misleading). Conversely, although heavy drinkers may well be under-represented in prospective studies, such studies have the great advantage that the information recorded at baseline about alcohol use cannot be distorted by the subsequent onset of disease. This allows unbiased comparison between the reported habits of those who do and do not die.

We report a large prospective study of self-reported alcohol (mainly vodka) consumption and mortality in the three Russian cities that we studied retrospectively. Since the number of deaths is smaller than that in the retrospective study, the analyses subdivide all-cause mortality into only two parts: those causes previously found to be alcohol-related (external causes and the eight disease groupings) and those not related to alcohol. The present analyses use the same age and vodka consumption categories as did the retrospective analyses, and hence test prospectively the retrospective findings;^10^ the two should be considered together.

## Methods

### Study design and participants

In this prospective study of mortality in 200 000 Russian adults, participants were recruited from three west Siberian industrial cities with predominantly European populations, and adult mortality rates similar to Russia as a whole:^10^ Barnaul (2002 population 0·7 million), Tomsk (0·5 million), and Byisk (0·2 million). Throughout 1999 (the first phase of recruitment, restricted to Barnaul and Tomsk), interviewers visited households randomly selected from electoral lists. During 2002–08 (the main phase of recruitment, including all three cities), interviewers visited households where an adult who had died several years ago (in 1990–2001) used to live, partly to ask surviving family members about the dead person for our retrospective study of alcohol use^10^ and partly to recruit adults still living there into the present prospective study.

Since almost all heavy drinkers were male smokers, our main analyses are of male smokers, although analyses of male non-smokers and women are also provided (appendix p 13). Results at ages 35–54 and 55–74 years are presented separately. We excluded people with no follow-up at 35–74 years, or with diseases that might alter drinking patterns (self-reported cancer, myocardial infarction, angina, heart failure, rheumatic heart disease, stroke, diabetes, tuberculosis, cirrhosis or chronic hepatitis), or who had stopped smoking or drinking due to illness (appendix pp 7–8).

Ethics approval was obtained from the WHO IARC Ethical Review Committee, the Research Ethics Committee of the Moscow Russian Cancer Research Centre, and the local research ethics committees.

### Procedures

In both phases, teams of local general practitioners known to the local population (and hence generally trusted by households) and trained in objective interview methods did the interviews. All registered residents of visited households who were at least 30 years old and were present when the household was visited were invited to join the study. Those who accepted answered questions (about smoking, drinking, education, work, and their history of serious illness), and had their blood pressure, height, weight, waist and hip circumference measured. Ex-smokers and ex-drinkers were asked whether they had stopped because of illness.

In 1999 the alcohol questions were only about current (ie, in the year before the baseline interview) consumption of vodka. In 2002–08, however, the questions were about both current and past consumption not only of vodka, but also of other strong alcoholic drinks, beer, and wine; the main source of alcohol was, however, vodka. Total consumption (frequency times amount, with beer taken as 0·125 and wine as 0·25 times vodka in strength) was described in units of half-litre bottles of vodka per week.^10^ The present analyses relate mortality only to current vodka consumption at baseline, and hence can include participants recruited in both phases.

Vodka consumption (in half-litre bottles per week) was divided into low (never drinkers, ex-drinkers who had not quit because of illness, and men drinking less than 1 bottle per week or women drinking less than 0·25 of a bottle per week), middle (men drinking 1 to <3 bottles per week or women drinking 0·25 to <1 bottle per week) and high (men drinking ≥3 bottles per week or women drinking ≥1 bottle per week). The appendix includes translations of the 1999 and of the 2002–08 questionnaires (pp 14–20). We noted a strong digit preference in the self-reported number of bottles of vodka per week, so we did not analyse consumption as a continuous variable.

Some 12 000 participants were interviewed twice (mainly because a participant could be interviewed both in the first and in the main phase). We used only the first interview in the prospective analyses of mortality; we used the second interview only to help assess the reproducibility of self-reported vodka consumption.

Long-term follow-up to Jan 1, 2010, was through local state mortality records, which include full name, address, date of birth, date of death and causes of death (underlying and proximal, in text and ICD-10 coded^12^) and are essentially complete. Record linkage was by a specially developed probabilistic algorithm, based on matching first name, father's name (traditionally used in Russia as an individual or personal identifier), second name, date of birth and address. It is unlikely that linkage on each of name, date of birth and address would be missed completely (and, a few missed linkages should not bias the findings appreciably). Where linkage was partial, additional checks by hand (occasionally including visits to the presumed decedent's address) were pursued until linkage was definitely confirmed or refuted. Causes of death were divided into those prespecified as alcohol-related (based on the retrospective study results) and those not; the appendix (p 10) gives the detailed ICD-10 codes.^12^

### Statistical analysis

For all-cause mortality and for the prespecified alcohol-related causes (with rates for other causes calculated as the difference), we calculated absolute death rates in the low, middle, and high alcohol groups in three stages. First, relative risks (RRs) were estimated by Poisson regression, adjusted for city, recruitment phase, attained age at risk (in 5-year bands), education (four levels), and cigarettes smoked per day (≤10, 11–19, 20, >20 cigarettes), all as categorical variables, with RR=1 for the high-alcohol group. Next, just for the high-alcohol group, we calculated in each 5-year age range the mortality rate as the number of deaths divided by person-years; we then defined the uniformly age-standardised rate in the age range 35–54 years (or, likewise, 55–74 years) as the average of the four age-specific rates within that age range. (If in a 20-year age range the uniformly age-standardised annual rate per 1000 persons is *R*, then exp[–20*R*/1000] is the conditional probability that someone who has survived to the start of that age range will survive to the end of it.) Finally, we multiplied these three RRs by the mortality rate in the high-alcohol group to obtain the mortality rates in each of the three alcohol groups, standardised to the pattern of risk factors in the high-alcohol group.

We used Plummer's method^13^ to estimate the variance of the log risk in each of the three alcohol groups. From these variances we derived 95% CIs that describe for the log risk in each of the three groups, including the reference group, the effects of the play of chance in the data just for that group. If, for a group with death rate *R*, the variance of the log risk is *v,* then the 95% CI for *R* runs from *R/k* to *R*×*k,* where *k*=exp(1·96√*v*). (In the case of only three groups, Plummer's method simplifies: if the log relative risks in the low-alcohol and middle-alcohol groups have respective variances *a* and *b* and covariance *c*, then the log risks in the low, middle, and high groups have respective variances *a*–*c, b*–*c* and *c*.)

To determine which diseases contributed most to the excess mortality among drinkers, we subcategorised underlying causes of death more finely, and for each subcategory we compared the numbers of deaths observed in the high-alcohol and middle-alcohol categories with the numbers expected at the age-specific death rates of the low-alcohol category (<1 bottle per week at baseline) in male smokers (appendix p 12).

Analyses used SASv9.3; figure-plotting used Rv3.0.

### Role of funding sources

The sponsors of the study had no role in study design, data collection, data analysis, data interpretation, or writing of the report. The corresponding authors had full access to all the data in the study and had final responsibility for the decision to submit for publication.

## Results

We obtained baseline interview data from 210 002 adults in 73 060 households. After exclusion of those too old or young to have any follow-up at ages 35–74 years, those with previous illness, and those who had stopped smoking or drinking because of illness (appendix pp 7–8), 151 811 remained for prospective follow-up of mortality.

Table 1 (also appendix p 9) shows the participants' characteristics, subdivided by their self-reported vodka consumption. Relatively few (compared with controls in our retrospective study)^10^ reported drinking one or more bottles per week of vodka: 19% (15 347 of 79 311) of the men and 2% (1209 of 72 500) of the women. Among them, intake of alcohol from other sources was only about a tenth that from vodka. Since intake of alcohol from other sources was much less than (and correlated with) intake from vodka, its consequences could not be studied.

**Table 1:** Characteristics and overall mortality of the 151 811 participants, by sex and vodka use self-reported at baseline*

	**Men**			**Women**		
	<1 half-litre bottle of vodka per week†	1 to <3 half-litre bottles of vodka per week	≥3 half-litre bottles of vodka per week	<0·25 of a half-litre bottle of vodka per week†	0·25 to <1 half-litre bottle of vodka per week	≥1 half-litre bottle of vodka per week
Number of interviewees	63 964	12 505	2842	67 288	4003	1209
Mean (SD) vodka consumption, bottles per week	0·2 (0·2)	1·3 (0·4)	4·9 (2·5)	0·0 (0·1)	0·4 (0·1)	2·0 (1·8)
Mean (SD) non-vodka consumption, units of 200 mL alcohol per week‡	0·2 (0·3)	0·3 (0·5)	0·5 (0·9)	0·1 (0·2)	0·2 (0·2)	0·3 (0·5)
Mean (SD) drinking frequency, days per week	0·8 (0·9)	1·8 (1·1)	4·2 (1·8)	0·3 (0·5)	1·1 (0·6)	2·5 (1·6)
Mean (SD) age, years	46·6 (11·3)	46·5 (10·0)	47·3 (9·8)	48·4 (12·0)	45·7 (10·0)	46·6 (10·4)
Never smoker	13 758 (21·5%)	1173 (9·4%)	198 (7·0%)	54 953 (81·7%)	2115 (52·8%)	415 (34·3%)
Ex-smoker§	6125 (9·6%)	594 (4·8%)	102 (3·6%)	2477 (3·7%)	241 (6·0%)	21 (1·7%)
Current smoker	44 081 (68·9%)	10 738 (85·9%)	2542 (89·4%)	9858 (14·7%)	1647 (41·1%)	773 (63·9%)
Mean (SD) number of cigarettes per day	16·4 (6·7)	19·1 (6·9)	21·3 (8·0)	9·3 (5·4)	11·2 (5·5)	13·4 (6·6)
Mean (SD) BMI, kg/m^2^¶	26·0 (3·4)	25·8 (3·5)	24·9 (3·5)	27·0 (4·6)	27·2 (4·6)	26·0 (5·0)
Mean (SD) SBP, mmHg¶	128 (15·4)	128 (16·0)	131 (17·4)	128 (19·2)	128 (18·4)	128 (19·4)
Mean (SD) DBP, mmHg¶	82 (8·9)	82 (9·3)	84 (10·3)	81 (10·9)	82 (10·8)	81 (11·7)
No education beyond primary school	3965 (6·2%)	1030 (8·2%)	437 (15·4%)	5834 (8·7%)	258 (6·4%)	201 (16·6%)
Manual worker	34 154 (53·4%)	8660 (69·3%)	1928 (67·8%)	15 723 (23·4%)	1348 (33·7%)	559 (46·2%)
Good cooperation‖	57 982 (90·6%)	10 769 (86·1%)	2638 (92·8%)	62 288 (92·6%)	3256 (81·3%)	1062 (87·8%)
Mean (SD) person-years at ages 35–74 years	5·2 (3·0)	5·5 (2·8)	6·3 (3·1)	6·3 (3·3)	5·2 (2·6)	6·3 (2·8)
Number of deaths at ages 35–74 years	3844	1052	516	2063	109	125

Data are n (%) unless otherwise specified. Mean consumption includes all drinks, and is expressed in units of 200 mL of pure alcohol per week (the approximate alcohol content of one bottle of vodka). BMI=body-mass index. SBP=systolic blood pressure. DBP=diastolic blood pressure.

* Excludes people with no follow-up at ages 35–74 years or with evidence at baseline of pre-existing disease (self-reported cancer, myocardial infarction, angina, heart failure, rheumatic heart disease, stroke, diabetes, tuberculosis, liver cirrhosis, or chronic hepatitis) or who had already quit drinking or smoking due to illness.

**Figure 1:**
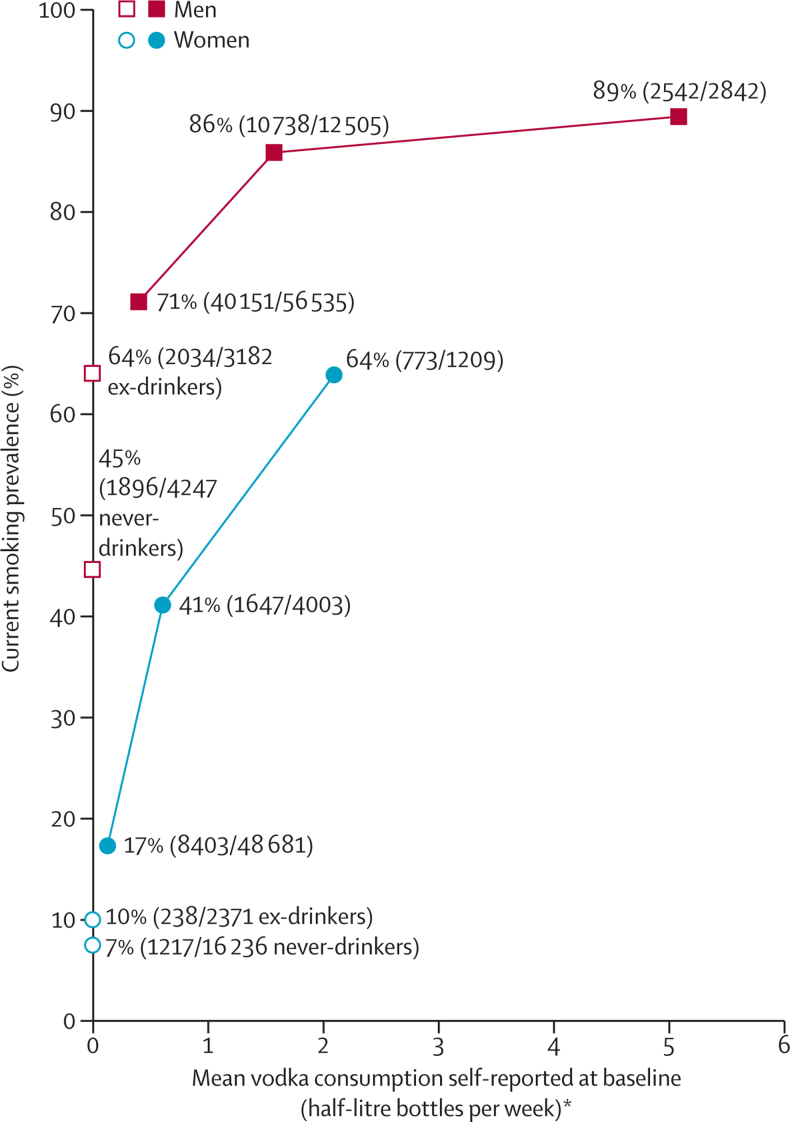
Prevalence of current smoking (%) in 151 811 participants versus mean vodka use self-reported at baseline Excludes people with no follow-up at ages 35–74 years or with evidence at baseline of pre-existing disease (self-reported cancer, myocardial infarction, angina, heart failure, rheumatic heart disease, stroke, diabetes, tuberculosis, liver cirrhosis, or chronic hepatitis) or who had already quit drinking or smoking due to illness. *Current drinkers were subdivided into prespecified categories of vodka consumption (for men <1, 1 to <3, ≥3 bottles per week, for women <0·25, 0·25 to <1, ≥1 bottle per week), but results are plotted against total consumption of alcohol, vodka or other, in units of 200 mL per week, as in table 1.

† Includes never-drinkers, ex-drinkers who did not quit because of illness, and low drinkers (<1 half-litre bottle of vodka per week for men or <0·25 of a half-litre bottle vodka per week for women): figure 1 separates never, ex and low·

‡ Phase 2 only (58 387 men, 41 738 women), no non-vodka consumption ascertained in phase 1.

§ Did not quit because of illness (see footnote *).

¶ Only 58 387 males and 41 738 females, as height, weight, and blood pressure were not measured during the first phase of recruitment.

‖ Good cooperation during interview, as assessed by interviewer.

Although vodka consumption correlated with manual work and lack of education (table 1), its main correlate was smoking (figure 1); moreover, mean cigarette consumption per smoker was greater in the high-vodka than in the low-vodka group.

Mean body-mass index (BMI) was slightly lower in the high- than in the low-vodka group, and in men (though not women) mean blood pressure was slightly higher (table 1). Our main mortality analyses do not adjust for these small differences. We rated cooperation with the baseline interview as having been good in about 90% of participants, largely irrespective of reported vodka consumption.

Self-reported vodka consumption can vary substantially over just a few years, increasing in some individuals and decreasing in others. Among the 6822 men who happened to be re-interviewed about 3 years after their baseline interview (table 2), 321 had reported drinking three or more bottles of vodka per week at baseline. When these heavy drinkers were re-interviewed, only 13% (41 of 321) still reported drinking three or more bottles per week, while 58% (185 of 321) reported drinking one bottle or less per week. Consequently, although their mean self-reported vodka consumption was 5·2 (SD 3·1) bottles per week at baseline, it was only 1·2 (SD 1·9) bottles per week at re-interview. Conversely, 14% (738 of 5435) of those who reported drinking less than one bottle per week at baseline reported drinking ≥1 bottle a week when re-interviewed about 3 years later. We noted similar patterns in women (appendix p 11).

**Table 2:** Vodka use self-reported at baseline and at unintended re-interview (mean 3 years later), 6822 men*

		**<1 half-litre bottle of vodka per week (n=5435)**	**1 to <3 half-litre bottles of vodka per week (n=1066)**	**≥3 half-litre bottles of vodka per week(n=321)**
Half-litre bottles of vodka per week reported at re-interview				
	<1	4697 (86%)	727 (68%)	185 (58%)
	1 to <3	653 (12%)	290 (27%)	95 (30%)
	≥3	85 (2%)	49 (5%)	41 (13%)
Mean (SD) vodka consumption reported at baseline, in units of 200 mL alcohol per week		0·1 (0·2)	1·3 (0·5)	5·2 (3·1)
Mean (SD) vodka consumption reported at re-interview, in units of 200 mL alcohol per week		0·4 (0·7)	0·7 (1·1)	1·2 (1·9)
Mean (SD) alcohol consumption reported at re-interview, in units of 200 mL alcohol per week		0·6 (0·9)	1·0 (1·1)	1·4 (2·1)

Data are n (%). Groupings in this table are defined only by vodka consumption, but mean alcohol consumption includes all drinks, and is expressed in units of 200 mL of pure alcohol per week (the approximate alcohol content of one bottle of vodka).

* 6822 men were unintentionally interviewed twice, generally once during phase 1 and once during phase 2 of recruitment; this number excludes men with no follow-up at ages 35–74 years, or with evidence at baseline (ie, at the first interview) of pre-existing disease (self-reported cancer, myocardial infarction, angina, heart failure, rheumatic heart disease, stroke, diabetes, tuberculosis, liver cirrhosis, or chronic hepatitis), or who at baseline had already quit drinking or smoking due to illness.

Of the 151 811 participants with no previous illness who were followed up for mortality at ages 35–74 years, 5412 of 79 311 men (7%) and 2297 of 72 500 women (3%) died in this age range. Of them, 2807 men and 782 women died from external causes and the eight disease groupings that had been prespecified as alcohol-related (introduction and appendix p 10). Table 3 gives for these causes, other causes, and all causes the numbers of deaths and the age-standardised death rates, according to vodka consumption at baseline. Results, subdivided by age (35–54 or 55–74 years), are given separately for male smokers, male non-smokers, female smokers, and female non-smokers. Appendix p 13 gives similar analyses, but with male ex-smokers and never-smokers subdivided.

**Table 3:** Mortality from causes prespecified as alcohol-related, other causes, and all causes, by sex, smoking habit at baseline, age at risk, and vodka use self-reported at baseline among 151 811 participants*

			**Prespecified as alcohol-related**		**Other causes**		**All causes**	
			Deaths	Annual rate per 1000 (95% CI)	Deaths	Annual rate per 1000 (95% CI)	Deaths	Annual rate per 1000 (95% CI)
**Male smokers (n=57 361)**								
Age at risk: 35–54 years								
	Never-drinker		38	4·9 (3·5–6·7)	21	2·8 (1·8–4·3)	59	7·7 (5·9–10·0)
	Ex-drinker†		47	6·2 (4·6–8·2)	20	2·5 (1·6–3·9)	67	8·7 (6·8–11·0)
		<1 half-litre bottle of vodka per week	675	5·5 (5·1–6·0)	339	3·1 (2·7–3·4)	1014	8·5 (8·0–9·1)
		1 to <3 half-litre bottles of vodka per week	313	8·1 (7·2–9·0)	107	3·0 (2·5–3·6)	420	11·0 (10·0–12·1)
		≥3 half-litre bottles of vodka per week	197	17·3 (14·9–20·0)	46	4·1 (3·0–5·5)	243	21·3 (18·7–24·3)
Age at risk: 55–74 years								
	Never-drinker		30	11·0 (7·7–15·9)	76	25·6 (20·4–32·2)	106	36·7 (30·2–44·5)
	Ex-drinker†		34	14·5 (10·3–20·3)	68	26·2 (20·6–33·3)	102	40·6 (33·4–49·4)
		<1 half-litre bottle of vodka per week	595	14·0 (12·8–15·2)	874	19·9 (18·5–21·3)	1469	33·8 (32·1–35·7)
		1 to <3 half-litre bottles of vodka per week	230	17·7 (15·5–20·1)	258	21·0 (18·6–23·7)	488	38·7 (35·4–42·3)
		≥3 half-litre bottles of vodka per week	115	26·3 (21·8–31·8)	100	24·8 (20·3–30·3)	215	51·1 (44·5–58·7)
**Male non-smokers (15 129 never-smokers, 6821 ex-smokers**†**)**								
Age at risk: 35–54 years								
	Never-drinker		17	2·2 (1·4–3·6)	20	3·1 (2·0–4·9)	37	5·3 (3·8–7·4)
	Ex-drinker†		9	3·2 (1·7–6·2)	3	1·2 (0·4–3·6)	12	4·4 (2·5–7·7)
		<1 half-litre bottle of vodka per week	135	3·3 (2·8–3·9)	81	2·3 (1·8–2·9)	216	5·6 (4·9–6·4)
		1 to <3 half-litre bottles of vodka per week	27	4·9 (3·3–7·2)	9	2·1 (1·1–4·1)	36	7·0 (5·1–9·8)
		≥3 half-litre bottles of vodka per week	18	18·8 (11·7–30·3)	4	5·4 (2·0–14·4)	22	24·2 (15·8–37·1)
Age at risk: 55–74 years								
	Never-drinker		31	5·4 (3·8–7·7)	75	14·2 (11·2–17·8)	106	19·5 (16·1–23·7)
	Ex-drinker†		19	8·1 (5·1–12·8)	31	13·7 (9·6–19·6)	50	21·8 (16·5–28·9)
		<1 half-litre bottle of vodka per week	201	6·3 (5·5–7·2)	405	14·1 (12·8–15·6)	606	20·4 (18·8–22·1)
		1 to <3 half-litre bottles of vodka per week	50	12·5 (9·5–16·6)	58	17·2 (13·3–22·3)	108	29·7 (24·6–36·0)
		≥3 half-litre bottles of vodka per week	26	24·6 (16·6–36·3)	10	12·0 (6·4–22·4)	36	36·6 (26·3–50·8)
**Female smokers (n=12 278)**								
Age at risk: 35–54 years								
	Never-drinker		16	3·7 (2·3–6·1)	15	3·1 (1·8–5·2)	31	6·8 (4·8–9·8)
	Ex-drinker†		2	2·8 (0·7–11·1)	3	4·2 (1·3–13·1)	5	6·9 (2·9–16·8)
		<0·25 of a half-litre bottle of vodka per week	74	3·5 (2·7–4·4)	51	2·2 (1·6–3·0)	125	5·7 (4·7–6·9)
		0·25 to <1 half-litre bottle of vodka per week	18	4·2 (2·6–6·7)	13	2·9 (1·7–5·1)	31	7·1 (5·0–10·2)
		≥1 half-litre bottle of vodka per week	32	8·9 (6·1–12·8)	6	1·6 (0·7–3·6)	38	10·5 (7·5–14·6)
Age at risk: 55–74 years								
	Never-drinker		8	8·3 (4·0–17·1)	18	13·1 (8·1–21·1)	26	21·4 (14·3–31·9)
	Ex-drinker†		2	8·4 (2·1–34·1)	7	26·9 (12·5–57·8)	9	35·2 (18·1–68·8)
		<0·25 of a half-litre bottle of vodka per week	28	7·7 (5·2–11·3)	63	14·6 (11·2–18·9)	91	22·3 (17·9–27·6)
		0·25 to <1 half-litre bottle of vodka per week	7	11·4 (5·3–24·5)	8	8·9 (4·4–17·9)	15	20·3 (12·1–34·0)
		≥1 half-litre bottle of vodka per week	24	32·9 (21·1–51·1)	11	12·0 (6·5–22·1)	35	44·8 (31·6–63·7)
**Female non-smokers (57 481 never-smokers, 2739 ex-smokers**†**)**								
Age at risk: 35–54 years								
	Never-drinker		24	0·6 (0·4–0·9)	68	1·5 (1·2–1·9)	92	2·1 (1·7–2·6)
	Ex-drinker†		3	1·0 (0·3–3·0)	4	1·2 (0·4–3·2)	7	2·2 (1·0–4·6)
		<0·25 of a half-litre bottle of vodka per week	137	1·1 (0·9–1·3)	158	1·1 (0·9–1·3)	295	2·2 (1·9–2·5)
		0·25 to <1 half-litre bottle of vodka per week	7	1·0 (0·5–2·2)	6	0·8 (0·3–1·7)	13	1·8 (1·0–3·1)
		≥1 half-litre bottle of vodka per week	10	6·3 (3·4–11·9)	3	1·5 (0·5–4·8)	13	7·9 (4·6–13·6)
Age at risk: 55–74 years								
	Never-drinker		131	2·4 (2·0–2·9)	437	8·0 (7·2–8·8)	568	10·4 (9·5–11·3)
	Ex-drinker†		19	3·2 (2·0–5·1)	51	7·4 (5·6–9·8)	70	10·6 (8·3–13·4)
		<0·25 of a half-litre bottle of vodka per week	190	2·1 (1·8–2·5)	554	6·8 (6·3–7·4)	744	9·0 (8·3–9·6)
		0·25 to <1 half-litre bottle of vodka per week	26	7·1 (4·8–10·6)	24	7·1 (4·7–10·6)	50	14·2 (10·8–18·8)
		≥1 half-litre bottle of vodka per week	24	20·1 (13·4–30·2)	15	15·2 (9·1–25·2)	39	35·3 (25·8–48·5)

Vodka use is given in half-litre bottles of vodka per week.

* Excludes people with no follow-up at ages 35–74 years or with evidence at baseline of pre-existing disease (self-reported cancer, myocardial infarction, angina, heart failure, rheumatic heart disease, stroke, diabetes, tuberculosis, liver cirrhosis, or chronic hepatitis), or who had already quit drinking or smoking due to illness.

† Did not quit due to illness.

There were too few deaths to determine reliably whether a little vodka consumption provided a slight protective effect or a slight hazard. In each of the eight combinations of age, sex, and smoking status, however, we noted a striking excess overall mortality in those who had reported drinking one or more bottles of vodka per week, with particularly high rates in those who had reported drinking three or more bottles per week. This excess overall mortality was driven by a strong association with mortality from those causes that had been prespecified^10^ as alcohol-related.

**Figure 2:**
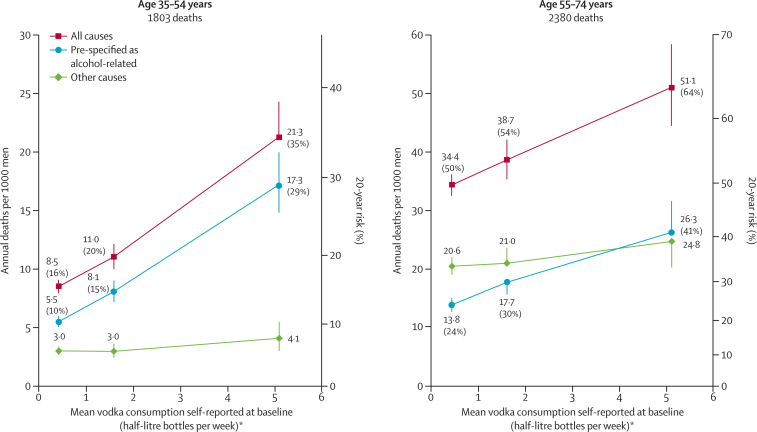
Mortality rates at ages 35–54 years and 55–74 years for causes prespecified as alcohol-related, other causes, and all causes, by vodka use self-reported at baseline in 57 361 male current smokers without previous disease The mortality rate in each 20-year age range is the mean of the four rates in the four 5-year age groups within that range, and the 20-year risk of death is conditional on reaching the start of the age range. Excludes people with no follow-up at ages 35–74 years or with evidence at baseline of pre-existing disease (self-reported cancer, myocardial infarction, angina, heart failure, rheumatic heart disease, stroke, diabetes, tuberculosis, liver cirrhosis, or chronic hepatitis) or who had already quit drinking or smoking because of illness. Causes prespecified as alcohol-related: external (includes assault, suicide, accident, alcohol poisoning, etc), liver diseases (neoplastic or not), upper aerodigestive cancer, tuberculosis, pneumonia, non-myocardial infarction acute ischaemic heart disease (ICD-10 I24), non-neoplastic pancreatic disease, and ill-defined causes. *Current drinkers were subdivided into prespecified categories of vodka consumption (for men <1, 1 to <3, ≥3 bottles per week), but results are plotted against total consumption of alcohol, vodka or other, in units of 200 mL per week, as in table 1.

Since most people who reported drinking one or more bottle per week at baseline were male smokers (figure 1), it was only in the male smokers that our mortality analyses were statistically stable. Figure 2 plots mortality at ages 35–54 and 55–74 years in male smokers versus baseline vodka consumption, combining never drinkers, ex-drinkers, and those consuming less than one bottle per week. These absolute death rates were adjusted for amount smoked (and other factors: see Methods) to the values in the high-alcohol group. Hence, the absolute mortality rates in the high-alcohol group are the crude age-standardised rates actually observed in that group.

In both age ranges vodka consumption was strongly related to overall mortality, mainly because of its relation to the causes of death that had been prespecified as alcohol-related. For male smokers of age 35–54 years, the uniformly age-standardised annual death rate per 1000 men was 21·3 (95% CI 18·7–24·3) in the highest vodka consumption category, compared with 8·5 (CI 8·0–9·1) in the lowest category; for male smokers of age 55–74 years, the corresponding rates in the highest and lowest vodka consumption categories were 51·1 (CI 44·5–58·7) and 34·4 (CI 32·6–36·3). These uniformly age-standardised annual death rates translate into 20-year mortality risks for high, middle and low self-reported vodka consumption of 35% (31–39), 20% (18–22), and 16% (15–17) at ages 35–54 years (trend p<0·0001), and 64% (59–69), 54% (51–57) and 50% (48–52) at ages 55–74 years (trend p<0·0001).

Figure 2 relates risk over a period of several years to self-reported consumption on just one occasion (ie, at the baseline survey). Table 2 showed, however, that many who described themselves as being in the high alcohol consumption category did not continue to drink heavily, whereas some who described themselves as being in the low alcohol consumption category later drank appreciably greater amounts of vodka. If, therefore, it had been possible to compare people who continued to drink heavily with people who never did so, the high-vodka group would have had even higher risks, the low-vodka group would have had even lower risks, and the relationship with mortality would have been much steeper than in figure 2.

The numbers of deaths from specific diseases and specific external causes were generally too small for similar analyses to yield statistically reliable absolute risks. Nevertheless, when underlying causes of death were subcategorised more finely (appendix p 12), the differences between the numbers observed among drinkers and the numbers expected at non-drinker death rates indicated that about half of the excess risk in heavy drinkers involved external causes, and that most of the remainder involved the somewhat strange ICD-10 category I24 (acute ischaemic heart disease that is not myocardial infarction).

## Discussion

This prospective study of alcohol and mortality in Russia provides strong, unbiased confirmation of the already striking findings from smaller prospective studies,^6^, ^11^ retrospective studies,^8^, ^10^ autopsy studies,^9^ and national mortality trends^2^, ^3^, ^4^, ^5^, ^7^, ^9^, ^10^ that vodka (or other strong alcoholic drink) is a major cause of death in Russia (panel).

Panel **Research in context**

**Systematic review**

Alcohol, particularly vodka, is a major cause of premature death in Russia. A large retrospective study^10^ suggested its main effects are on mortality from external causes and eight particular disease groupings. We searched in PubMed using the search words (“Russia”) AND (“alcohol” OR “vodka”) AND (“death” OR “mortality”), for articles in English published before 18 October 2013, but found only retrospective studies and small prospective studies. The present large prospective study now confirms vodka use is strongly predictive of premature death, mainly from causes expected from the findings of the large retrospective study.

**Interpretation**

Studies of national mortality trends, retrospective studies, and large prospective studies have complementary strengths, and with the present study now reinforce each other^2^, ^3^, ^4^, ^5^, ^6^, ^7^, ^8^, ^9^, ^10^ as evidence that the extraordinarily high risk of premature death among Russian adults, particularly men, is chiefly due to the use of vodka and other strong alcoholic drink. As almost all Russians who drink more than one bottle of vodka a week also smoke, any studies of the hazards of drinking have to be largely restricted to its effects among smokers, and any studies of the hazards of smoking will have to be largely restricted to its effects in those drinking less than one bottle of vodka a week.

**Figure 3:**
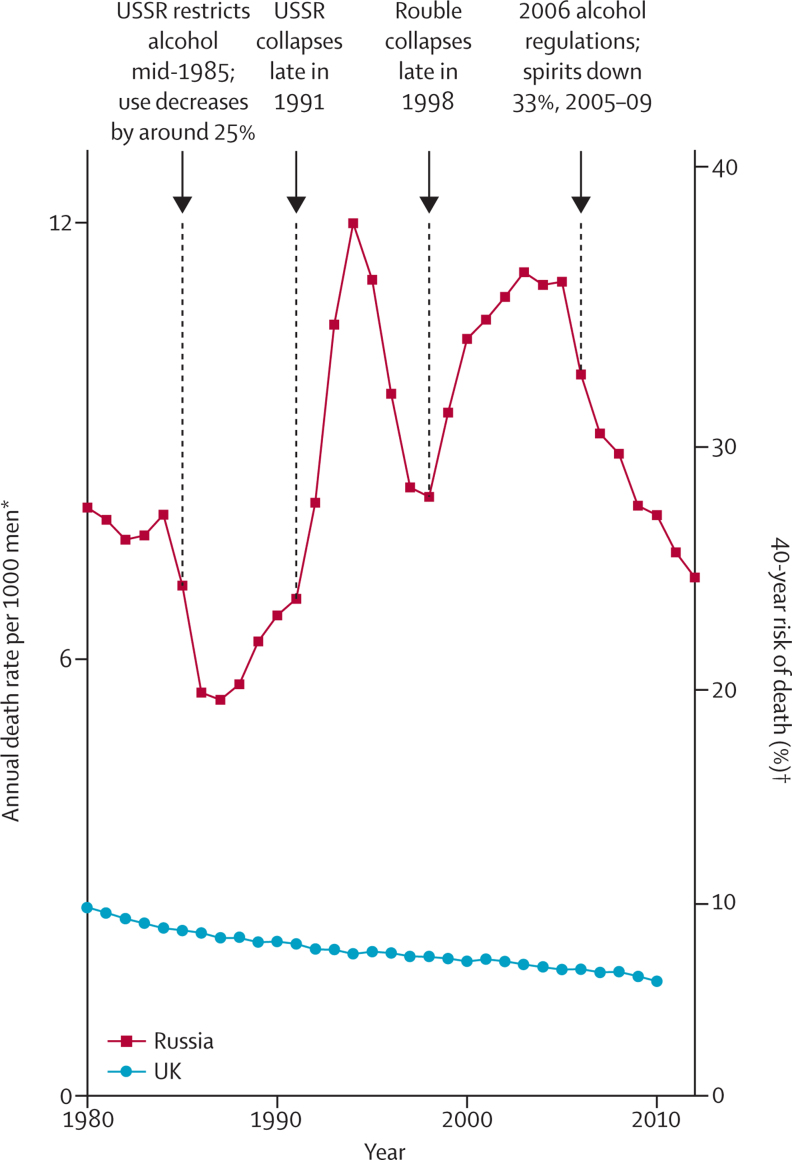
All-cause mortality, males aged 15–54 years, in Russia from 1980–2012 and in the UK from 1980–2010 *Mean of male age-specific death rates in the eight component 5-year age groups (15–19 to 50–54 years). †Probability that a 15-year-old male individual would die before age 55 years, if exposed over next 40 years to male age-specific death rates of one particular calendar year.

A retrospective study of 50 000 deaths in the same cities^10^ had found a marked excess of heavy vodka use in those whose death was attributed to external causes (accident, suicide, violence, and alcohol poisoning) or eight particular disease groupings (cancer of the upper aerodigestive tract, tuberculosis, pneumonia, liver cancer, other liver disease, pancreatic disease, acute ischaemic heart disease that is not myocardial infarction (I24), and ill-specified disease). Sharp fluctuations in Russian mortality rates from these causes during the 1990s were the main reason for the sudden large fluctuations in premature mortality in women and, particularly, men (figure 3; appendix pp 3–5).^9^, ^10^

It is unclear what medical conditions predominate among the Russian deaths ascribed to the ICD-10 category I24 (used commonly in Russia but rarely in the UK), yet I24 dominates the high level and sharp fluctuations of Russian mortality from supposedly vascular causes.^9^ Many whose deaths are ascribed to it have high post-mortem alcohol concentrations, so it probably includes some deaths from acute effects of alcohol, perhaps superposed on chronic effects.^9^

Retrospective studies of alcohol and mortality seek a family member or other informant who can describe the alcohol consumption of the dead person. They have the great advantage that they can begin with a reasonably representative sample of all deaths and can study large numbers of deaths quickly, but could be subject to two sources of bias. First, if the dead person had become socially isolated because of heavy drinking it might be difficult to find a suitable informant. Second, if the informant thought the person had died because of drink then this knowledge might bias their description of the dead person's drinking habits.

Prospective studies avoid these disadvantages, but take longer to observe large numbers of deaths (partly because those at high risk of death might not join such a study, and because, to avoid reverse causality, the analyses exclude those with prior disease). More than a decade since it began, our prospective study of 200 000 adults has accumulated only 8000 deaths at ages 35–74 years among the 151 000 participants with no previous disease at baseline and some follow-up in this age range. This is not enough to yield reliable dose-response results for each separate disease. To avoid unduly data-dependent groupings we therefore divided underlying causes into only two categories: those prespecified from the retrospective study findings^10^ as strongly alcohol-related, and all others.

Likewise, we used the same alcohol consumption categories as the retrospective study.^10^ Adjustment for our measures of amount smoked, educational and social factors made little difference to the hazards associated with alcohol, so major residual confounding by such factors is unlikely, and adjustment for recruitment phase allows for any differences in questionnaire design (appendix pp 14–20) or in drinking prevalence. Alcohol intake from vodka (sold legally or illegally) predominated. Information was not sought on use of illicit drugs or on surrogate (eg, industrial or medicinal^8^) ethanol products that are not taxable as alcoholic drinks.

One limitation of our prospective study is that most heavy drinkers were male smokers, so our main description of the effects of heavy drinking is restricted to male smokers (although such information as we have suggests that females and non-smokers would have similar absolute risks from drinking heavily if they were to do so). Another is that heavy drinkers are under-represented, so this study cannot directly estimate the alcohol-attributed fraction of overall mortality in Russia. (The proportions of men and women reportedly drinking at least one bottle of vodka a week were only 19% and 2% in the present study, as against 47% and 11% among the controls in our retrospective study in the same three cities.^10^)

The main limitation, however, is that self-described drinking habits are inaccurate, and in addition a resurvey showed that they can vary greatly over just a few years (as can Russian consumption of spirits,^14^, ^15^ appendix p 6). Hence, even the extreme differences in overall mortality that we found (eg, figure 2, table 3) substantially under-estimate the real hazards of prolonged heavy consumption of vodka (or other strong alcoholic drinks) in Russia. Exact allowance for the effects of such inaccuracies is impossible, but even approximate allowance for them would make the relative risks for death from external causes or from the eight alcohol-related disease groupings substantially steeper than those in figure 2, and hence more closely consistent with the extreme relative risks noted in the retrospective study.^10^ Thus, our findings strongly reinforce the past and present findings from the Global Burden of Disease project^16^, ^17^ about the importance of alcohol as a cause of death.

Nevertheless, even within the relatively low-risk circumstances of a prospective study, we found large differences in overall mortality between the top and bottom alcohol consumption categories that were driven mainly by the causes prespecified as alcohol-related. This provides strong confirmation, free from the disadvantages of retrospective mortality analyses, that alcohol, particularly vodka, is a major determinant of mortality from these causes, and hence of the high and sharply fluctuating Russian national mortality rates.

**This online publication has been corrected. The corrected version first appeared at thelancet.com on April 25, 2014**
