# Supplementary material for: Alcohol and mortality in Russia: prospective observational study of 151 000 adults
Source: Lancet. 2014 Apr 26;383(9927):1465–73. doi: 10.1016/S0140-6736(13)62247-3 (PMC4007591; doi:10.1016/S0140-6736(13)62247-3)
Supplement: Supplementary appendix [file mmc1.pdf]

THE LANCET

Supplementary appendix

This appendix formed part of the original submission and has been peer reviewed. We post it as supplied by the authors.

Supplement to: Zaridze D, Lewington S, Boroda A, et al. Alcohol and mortality in Russia: prospective observational study of 151 000 adults. *Lancet* 2014; published online Jan 31. http://dx.doi.org/10.1016/S0140-6736(13)62247-3.

Russian Prospective Study
Contents list of Appendix

p 3: Male all-cause mortality at ages 15-34, 35-54 and 55-74, Russia 1980-2012 and UK

p 4: Female all-cause mortality at ages 15-34, 35-54 and 55-74, Russia 1980-2012 and UK

p 5: Male and female all-cause mortality at ages 15-54, Russia 1980-2012 and UK

p 6: 2000-2012 Russian alcohol sales (litres alcohol/adult)

p 7: Number of interviewees by place, sex, recruitment phase and age at baseline

p 8: Smoking and disease history by drinking status at baseline for the 197 493 interviewees with some follow-up during ages 35-74 years (of whom 151 811 participants contribute to the main analyses)

p 9: Characteristics of the study population by sex and drinking status self-reported at baseline for 151 811 participants

p 10: ICD-10 codes and numbers of deaths at ages 35-74 years, for specific causes among 151 811 participants

p 11: Vodka use self-reported at baseline and at unintended re-interview (mean 2.9 years later): 5192 women

p 12: Observed and expected numbers of deaths from specific causes or groups of causes, by vodka use self-reported at baseline in 57 361 male smokers

p 13: Mortality from causes pre-specified as alcohol-related, other causes, and all causes, by sex, smoking habit at baseline, age at risk and vodka use self-reported at baseline among 151 811 participants

pp 14-15: 2-page baseline questionnaire* (for phase 1 of recruitment,

Jan-Dec 1999)

pp 16-20: 5-page baseline questionnaire* (for phase 2 of recruitment, 2002-08)

* English translation of Russian original

Males, Total mortality
Russia 1980−2012 and UK (to 2010)

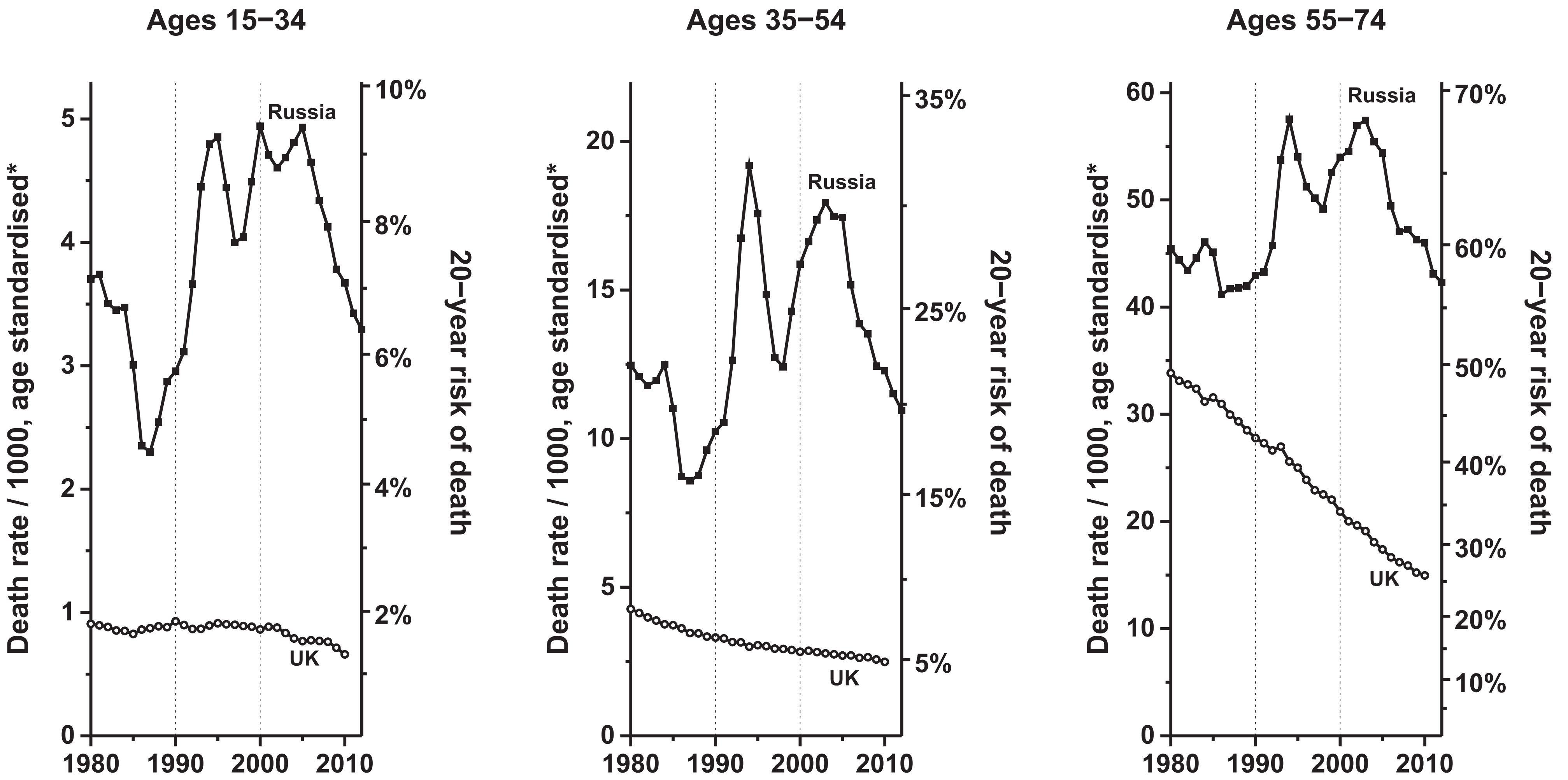

*Mean of annual rates in four 5−year age groups

Source: WHO (& ZAGS 2011−12) mortality and UN population estimates

Females, Total mortality
Russia 1980−2012 and UK (to 2010)

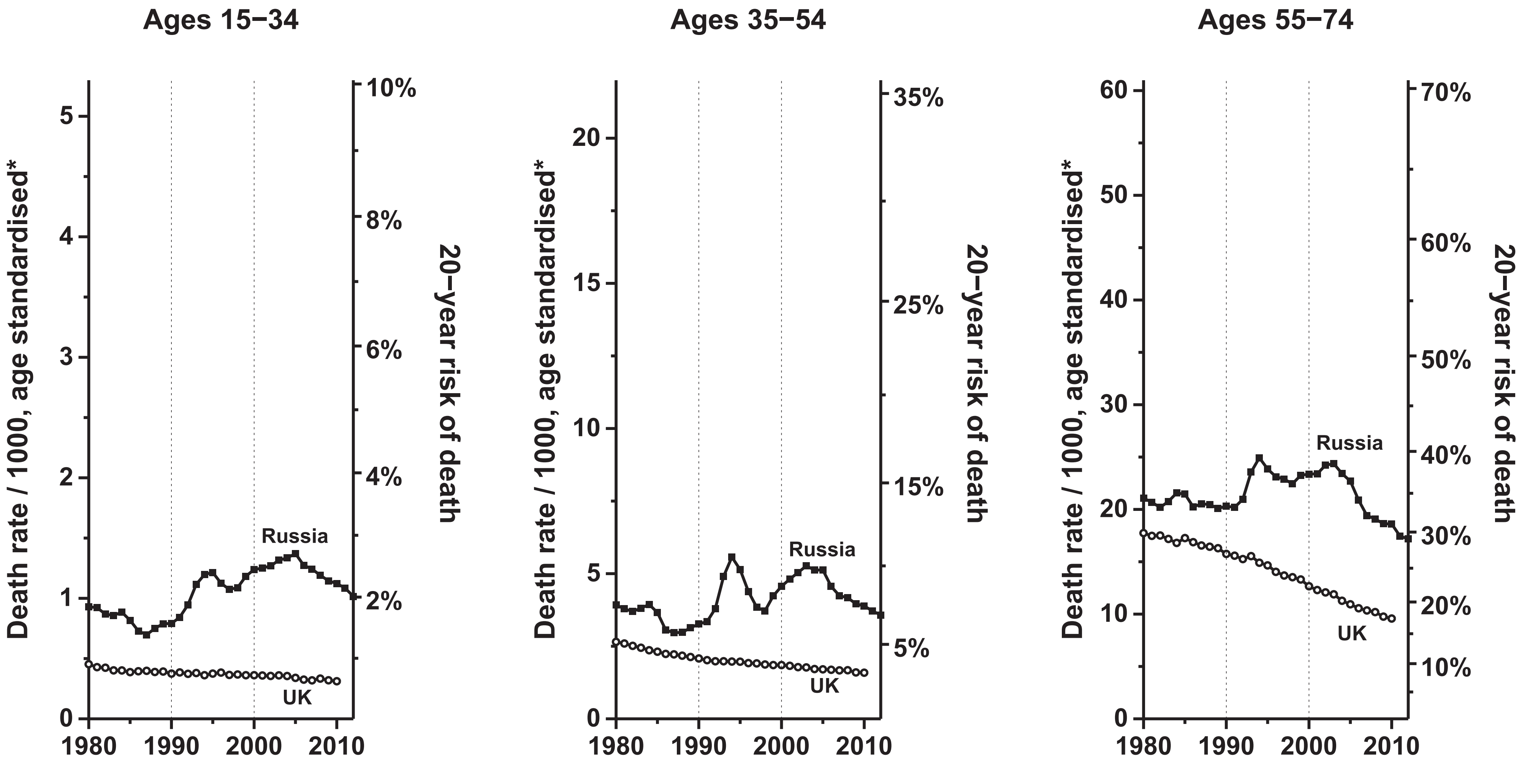

*Mean of annual rates in four 5−year age groups

Source: WHO (& ZAGS 2011−12) mortality and UN population estimates

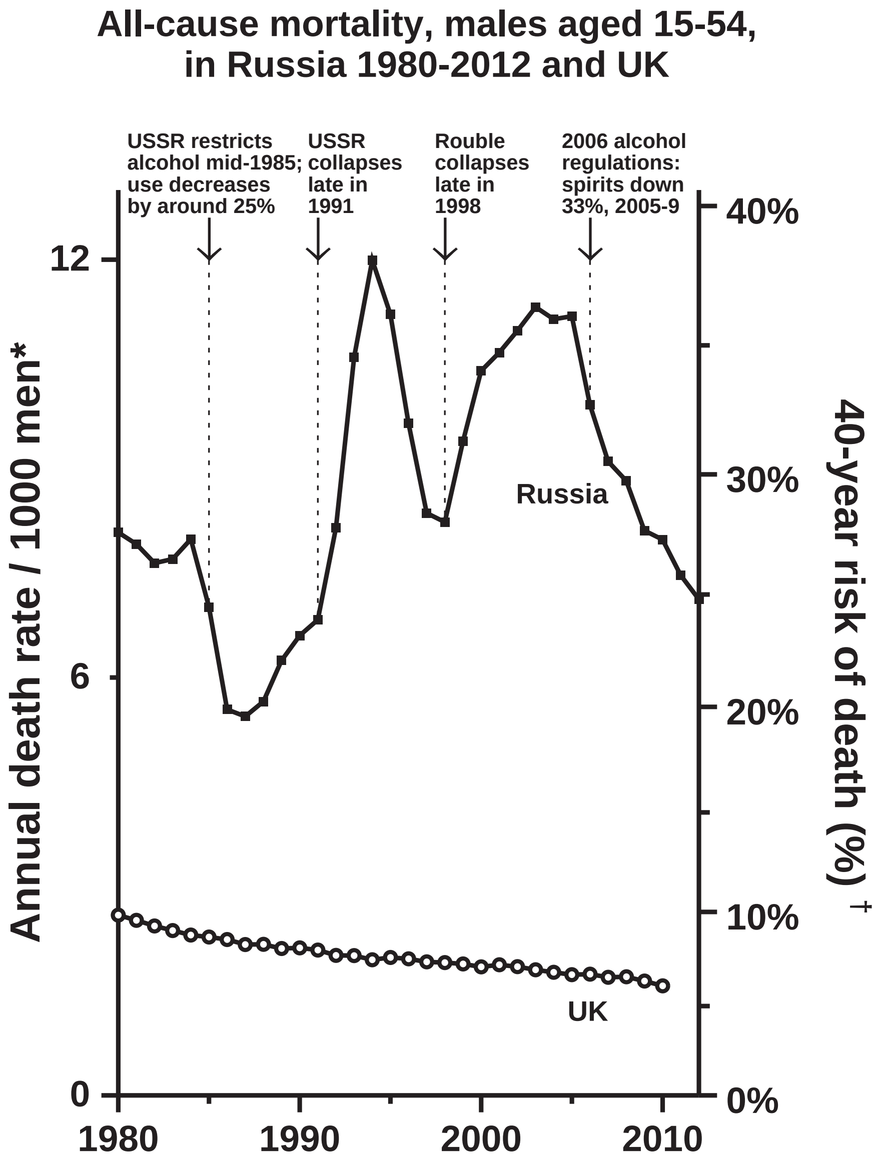

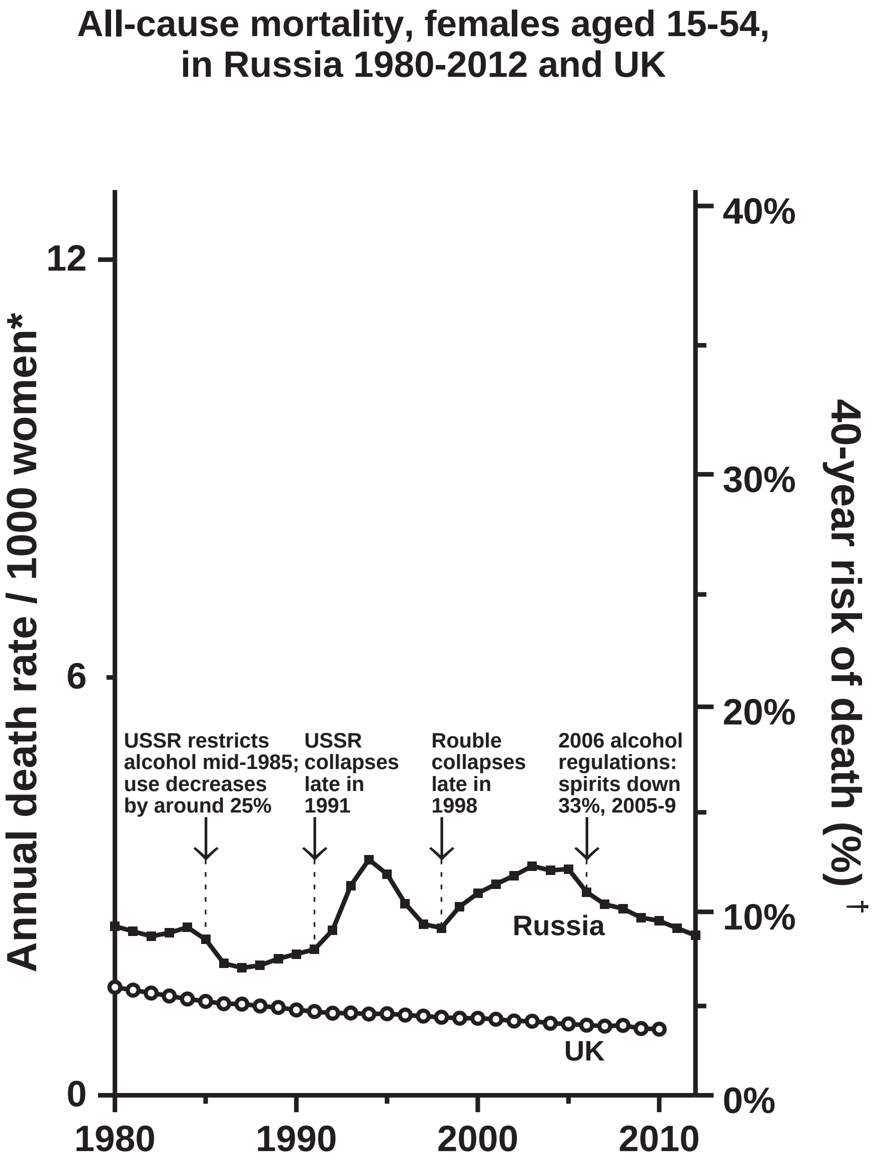

Source: WHO mortality & UN population estimates

*** Mean of age-specific death rates in the eight component 5-year age groups (15-19 to 50-54)**

† Probability a 15-year-old would die before age 55, if exposed over next 40 years to age-specific death rates of one particular calendar year

2000-2012 Russian alcohol sales (litres alcohol/adult), including Rosstat estimates of unrecorded spirit sales *						
**Year**	**Total**	**Beer**	**Wine**	**Spirits recorded**	**Spirits, not recorded**	**Total spirits**
2000	16.64	2.17	0.70	7.25	6.52	13.77
2001	17.40	2.62	0.74	7.05	6.99	14.04
2002	17.28	2.91	0.79	7.11	6.47	13.58
2003	17.44	3.08	0.89	7.28	6.18	13.46
2004	16.93	3.45	0.94	7.06	5.47	12.53
2005	16.42	3.65	1.02	6.88	4.86	11.74
2006 †	15.68	4.11	1.00	6.72	3.85	10.57
2007	14.98	4.75	1.18	6.37	2.69	9.06
2008	14.55	4.68	1.27	6.19	2.40	8.59
2009	13.65	4.22	1.26	5.82	2.35	8.17
2010	13.54	4.21	1.31	5.66	2.35	8.01
2011	13.16	4.11	1.23	5.47	2.35	7.82
2012	13.55	4.32	1.23	5.64	2.35	7.99

*Source: Russian government website (accessed Oct, 15 2013)
www.gks.ru/wps/wcm/connect/rosstat_main/rosstat/en/main/

† Regulation of alcohol sales was strengthened in 2006:
Neufeld M, Rehm J. Alcohol consumption and mortality in Russia since 2000 – are there any changes following the alcohol policy changes starting in 2006? *Alcohol and alcoholism* 2013; **48:** 222-30.

Appendix p 7: Number of interviewees by place, sex, recruitment phase and age at baseline

		Number of interviewees								
		Male				Female				
Recruitment phase (and year)	Place	<35 y[a]	35-54 y	55-74 y	75+ y	<35 y[a]	35-54 y	55-74 y	75+ y	Total
Phase 1 (1999)	Barnaul	1710	8308	5345	1000	2249	11221	9446	2957	42236
	Tomsk	1181	6247	3924	708	1535	8463	6432	1931	30421
Phase 2 (2002-08)	Barnaul	7095	19181	12259	0	4267	11444	9784	0	64030
	Byisk	1793	5851	3320	0	709	2648	2535	0	16856
	Tomsk	5877	15609	8727	0	3885	13440	8921	0	56459
Total		17656	55196	33575	1708	12645	47216	37118	4888	210002

[a] 24 388 of those aged <35 years at baseline had some follow-up at ages 35-74 years, so total with some follow-up at ages 35-74 years is 197 493.

Appendix p 8: Smoking and disease history by drinking status at baseline for the 197 493 interviewees with some follow-up during ages 35-74 years (of whom 151 811 participants contribute to the main analyses[a])

	Number of interviewees									
	Male					Female				
	Never-drinker	Ex-drinker (had quit because of illness)[a]	Other ex-drinker	Current drinker	Total	Never-drinker	Ex-drinker (had quit because of illness)[a]	Other ex-drinker	Current drinker	Total
Self-reported history of a disease that caused exclusion[a]	1454	3246	861	13796	19357	6599	2809	1148	10118	20674
No self-reported history of specific disease, but had quit smoking because of illness[a]	158	342	74	1823	2397	72	55	13	306	446
All others	**4247**[b]	1342	**3182**[b]	**71882**[b]	80653	**16236**[b]	1466	**2371**[b]	**53893**[b]	73966
Total	5859	4930	4117	87501	102407	22907	4330	3532	64317	95086

[a] People with a self-reported history of cancer, MI, angina, heart failure, rheumatic heart disease, stroke, diabetes, TB, liver cirrhosis or chronic hepatitis were excluded from the main analyses, as were those who had stopped drinking or smoking because of illness.

[b] These six groups are the 151 811 participants (79 311 male, 72 500 female) who contribute to the main analyses.

Appendix p 9: Characteristics of the study population by sex and drinking status self-reported at baseline for 151 811 participants[a]

	Male			Female		
	**Never-drinker**	**Ex-drinker**	**Current drinker**	**Never-drinker**	**Ex-drinker**	**Current drinker**
Number of interviewees	4247	3182	71882	16236	2371	53893
Mean (SD) age, years	46.9 (12.1)	49.0 (11.5)	46.5 (11.0)	51.5 (12.9)	55.1 (13.3)	46.9 (11.2)
Never smoker, n (%)	1959 (46.1)	605 (19.0)	12565 (17.5)	14694 (90.5)	2026 (85.4)	40763 (75.6)
Ex-smoker, n (%)	392 (9.2)	543 (17.1)	5886 (8.2)	325 (2.0)	107 (4.5)	2307 (4.3)
Current smoker, n (%)	1896 (44.6)	2034 (63.9)	53431 (74.3)	1217 (7.5)	238 (10.0)	10823 (20.1)
Mean (SD) number of cigarettes/day	15.9 (7.3)	19.0 (8.1)	17.1 (6.9)	9.8 (6.4)	11.4 (6.7)	9.8 (5.5)
Mean (SD) BMI[b], kg/m^2	25.7 (3.7)	25.6 (3.7)	25.9 (3.4)	26.8 (4.8)	27.4 (4.9)	27.0 (4.5)
Mean (SD) SBP[b], mmHg	128 (17.2)	128 (15.7)	128 (15.5)	130 (21.0)	134 (22.2)	127 (18.4)
Mean (SD) DBP[b], mmHg	82 (10.1)	82 (9.3)	82 (8.9)	82 (11.3)	84 (11.7)	81 (10.7)
No education beyond primary school, n (%)	446 (10.5)	381 (12.0)	4605 (6.4)	2528 (15.6)	426 (18.0)	3339 (6.2)
Manual worker, n (%)	1847 (43.5)	2123 (66.7)	40772 (56.7)	3502 (21.6)	796 (33.6)	13332 (24.7)
Good cooperation with interview[c], n (%)	3902 (91.9)	2924 (91.9)	64563 (89.8)	15007 (92.4)	2140 (90.3)	49459 (91.8)

[a] Excludes people with no follow-up at ages 35-74 years or with evidence at baseline of pre-existing disease (self-reported cancer, myocardial infacton (MI), angina, heart failure, rheumatic heart disease, stroke, diabetes, tuberculosis (TB), liver cirrhosis or chronic hepatitis), or who had already quit drinking or smoking due to illness.

[b] Only 58 387 males and 41 738 females, as height, weight and blood pressure were not measured during the first phase of recruitment.

[c] As assessed by interviewer.

Appendix p 10: ICD-10 codes and numbers of deaths at ages 35-74 years, for specific causes of death among 151 811[a] participants

Cause of death	ICD-10 code(s)	Number of deaths Male (n=79 311)	Female (n=72 500)
**Pre-specified alcohol-related**	Sum of components below	2807	782
External causes	F10, S00-T99, V00-Y99	966	243
Alcohol poisoning	F10, T51, X45, Y15	92	17
Suicide	X60-84	133	26
Assault	X85-Y09, Y35-36	92	23
Transport accidents	V00-99	47	25
Other external	Remainder of S00-T99, W00-Y99	602	152
Liver cancer	C22	40	24
Other liver disease[b]	B15-19, K70-77, I85	128	87
Upper aerodigestive cancer	C00-15, 32	121	9
Tuberculosis	A15-19, B90	110	8
Pneumonia, etc[b]	J00-39, J60-98	223	73
Non-MI acute IHD	I24	824	217
Non-neoplastic pancreatic disease	K85-86	50	21
Ill-specified disease	R00-99	345	100
**Other vascular diseases**	Sum of components below	1258	703
Acute MI or angina	I20-23	205	87
Chronic IHD	I25	308	134
Stroke	I60-69	501	382
Other vascular disease	I00-99 except I20-25, 27, 60-69, 85	244	100
**Other neoplastic diseases**	Sum of components below	842	471
Lung cancer	C33-34	389	39
Breast cancer	C50	2	112
Colorectal cancer	C18-21, 26	105	105
Pancreatic cancer	C25	54	30
Stomach cancer	C16	150	70
Cancer site unspecified	C76-80	35	25
Other neoplastic diseases	Remainder of C00-99, D00-48	107	90
**Other diseases**	Sum of components below	505	341
Gastro-oesophageal disease[b]	K20-31, 92	45	21
Peritonitis	K65	7	4
COPD[b]	I27, J40-47	110	27
Other specifed disease	Any remaining causes	343	289
**All causes**	A00-T98, V01-Z99	5412	2297

n = number of risk.

COPD=chronic obstructive pulmonary disease; IHD=ischaemic heart disease; MI=myocardial infarction.

[a] Excludes people with no follow-up at ages 35-74 years or with evidence at baseline of pre-existing disease (self-reported cancer, myocardial infarction (MI), angina, heart failure, rheumatic heart disease, stroke, diabetes, tuberculosis (TB), liver cirrhosis or chronic hepatitis), or who had already quit drinking or smoking due to illness.

[b] Differences compared with the 2009 report (and numbers of deaths affected): I85 (oesophageal varices, 4 deaths) moved from gastro-oesophageal disease to other liver disease and J67 (hypersensitivity pneumonitis, 0 deaths) moved from COPD to other respiratory.

Appendix p 11: Vodka use self-reported at baseline and at unintended re-interview (mean 2.9 years later): 5192 women[a]

Groupings in this table are defined only by vodka consumption, but mean alcohol consumption includes all drinks, and is expressed in units of 200g of pure alcohol per week (the approximate alcohol content of one bottle of vodka)

		Number (%)		
		Half-litre bottles of vodka/week reported at baseline		
		<¼<br>n = 4935	¼ to <1<br>n = 187	≥1<br>n = 70
**Half-litre bottles of vodka/week reported at re-interview**	**<¼**	4658 (94%)	144 (77%)	42 (60%)
	**¼ to <1**	230 (5%)	37 (20%)	7 (10%)
	**≥1**	47 (1%)	6 (3%)	21 (30%)
Mean (SD) vodka consumption reported at baseline, in units of 200g alcohol/week		0.0 (0.0)	0.4 (0.1)	1.6 (1.1)
Mean (SD) vodka consumption reported at re-interview, in units of 200g alcohol/week		0.1 (0.2)	0.2 (0.3)	0.8 (1.4)
Mean (SD) alcohol consumption reported at re-interview, in units of 200g alcohol/week		0.2 (0.3)	0.4 (0.4)	1.0 (1.3)

[a] 5192 women were unintentionally interviewed twice, generally once during phase 1 and once during phase 2 of recruitment. This number excludes women with no follow-up at ages 35-74 years, or with evidence at baseline (ie, at the first interview) of pre-existing disease (self-reported cancer, MI, angina, heart failure, rheumatic heart disease, stroke, diabetes, TB, liver cirrhosis, chronic hepatitis), or who at baseline had already quit drinking or smoking due to illness.

Appendix p 12: Observed and expected numbers of deaths from specific causes or groups of causes, by vodka use self-reported at baseline in 57 361 male smokers[a]
(Expected numbers are calculated by applying the age-specific rates among those who reported drinking <1 bottle vodka/week[b])

	35-54 years						55-74 years					
	1-<3 half-litre bottles vodka/week			3+ half-litre bottles vodka/week			1-<3 half-litre bottles vodka/week			3+ half-litre bottles vodka/week		
Cause of death	Observed	Expected	O/E	Observed	Expected	O/E	Observed	Expected	O/E	Observed	Expected	O/E
**Pre-specified as alcohol-related**	**313**	**195.0**	**1.6**	**197**	**54.1**	**3.6**	**230**	**169.1**	**1.4**	**115**	**46.9**	**2.5**
External causes	138	79.6	1.7	67	22.1	3.0	52	42.1	1.2	34	11.7	2.9
Alcohol poisoning	14	7.7	1.8	10	2.1	4.7	1	2.6	0.4	3	0.7	4.2
Suicide	15	12.8	1.2	8	3.6	2.2	9	6.9	1.3	2	1.9	1.0
Assault	13	8.2	1.6	8	2.3	3.5	7	4.4	1.6	2	1.2	1.7
Transport accidents	8	3.6	2.2	1	1.0	1.0	5	1.3	3.9	0	0.4	0.0
Other external	88	47.2	1.9	40	13.1	3.1	30	26.9	1.1	27	7.5	3.6
Liver cancer	0	1.0	0.0	0	0.3	0.0	3	5.1	0.6	1	1.4	0.7
Other liver disease	7	10.0	0.7	9	2.8	3.2	8	6.7	1.2	6	1.9	3.2
Upper aerodigestive cancer	8	4.9	1.6	6	1.4	4.4	16	13.6	1.2	4	3.8	1.1
Tuberculosis	20	8.2	2.4	15	2.3	6.6	10	6.9	1.4	2	1.9	1.0
Pneumonia, etc	24	13.1	1.8	22	3.6	6.1	24	16.9	1.4	10	4.7	2.1
Non-MI acute IHD	76	47.5	1.6	44	13.2	3.3	91	58.0	1.6	42	16.1	2.6
Non-neoplastic pancreatic disease	3	5.4	0.6	7	1.5	4.7	1	1.3	0.8	3	0.4	8.3
Ill-specified disease	37	25.4	1.5	27	7.0	3.8	25	18.5	1.4	13	5.1	2.5
**Other vascular diseases**	63	45.4	1.4	21	12.6	1.7	114	121.4	0.9	47	33.6	1.4
Acute MI or angina	12	8.7	1.4	4	2.4	1.7	16	21.3	0.8	9	5.9	1.5
Chronic IHD	9	8.7	1.0	3	2.4	1.2	27	34.9	0.8	14	9.7	1.4
Stroke	19	15.4	1.2	8	4.3	1.9	52	48.2	1.1	18	13.4	1.3
Other vascular disease	23	12.6	1.8	6	3.5	1.7	19	16.9	1.1	6	4.7	1.3
**Other neoplastic diseases**	24	32.1	0.7	14	8.9	1.6	100	88.0	1.1	29	24.4	1.2
Lung cancer	8	14.6	0.5	7	4.1	1.7	52	50.8	1.0	16	14.1	1.1
Breast cancer	1	0.0	-	0	0.0	-	0	0.0	-	0	0.0	-
Colorectal cancer	3	3.9	0.8	1	1.1	0.9	12	7.4	1.6	3	2.1	1.5
Pancreatic cancer	3	1.3	2.3	0	0.4	0.0	6	4.6	1.3	4	1.3	3.1
Stomach cancer	4	6.2	0.6	3	1.7	1.8	19	12.3	1.5	3	3.4	0.9
Cancer site unspecified	1	2.1	0.5	2	0.6	3.5	5	3.1	1.6	1	0.9	1.2
Other neoplastic diseases	4	4.1	1.0	1	1.1	0.9	6	9.8	0.6	2	2.7	0.7
**Other diseases**	20	20.0	1.0	11	5.6	2.0	44	51.8	0.8	24	14.4	1.7
Gastro-oesophageal disease	4	2.6	1.6	1	0.7	1.4	1	4.6	0.2	2	1.3	1.6
Peritonitis	0	0.3	0.0	1	0.1	14.3	1	0.3	3.8	1	0.1	14.3
COPD	4	2.3	1.7	1	0.6	1.6	10	16.2	0.6	6	4.5	1.3
Other specified disease	12	14.9	0.8	8	4.1	1.9	32	30.8	1.0	15	8.5	1.8
**All causes**	**420**	**292.5**	**1.4**	**243**	**81.1**	**3.0**	**488**	**430.3**	**1.1**	**215**	**119.3**	**1.8**

[a] Excludes people with no follow-up at ages 35-74 years or with evidence of pre-existing disease (self-reported cancer, MI, angina, heart failure, rheumatic heart disease, stroke, diabetes, TB, liver cirrhosis or chronic hepatitis) or quit drinking or smoking due to illness.
[b] Never-drinkers, ex-drinkers who did not quit because of illness and current low drinkers (<1 bottle of vodka or equivalent/week).

Appendix p 13: Mortality from causes pre-specified as alcohol-related, other causes, and all causes, by sex, smoking habit at baseline, age at risk and vodka use self-reported at baseline among 151 811 participants[a]
Omits the few (2739/151 811) female ex-smokers in Table 3. Vodka use is given in half-litre bottles of vodka per week.

	Pre-specified as alcohol-related		Other causes		All causes	
	Deaths	Annual rate/1000 (95% CI)	Deaths	Annual rate/1000 (95% CI)	Deaths	Annual rate/1000 (95% CI)
**Male smokers (n=57 361)**						
Age at risk: 35-54						
Never-drinker	38	4.9 (3.5-6.7)	21	2.8 (1.8-4.3)	59	7.7 (5.9-10.0)
Ex-drinker[b]	47	6.2 (4.6-8.2)	20	2.5 (1.6-3.9)	67	8.7 (6.8-11.0)
<1[c]	675	5.5 (5.1-6.0)	339	3.1 (2.7-3.4)	1014	8.5 (8.0-9.1)
1 to <3[c]	313	8.1 (7.2-9.0)	107	3.0 (2.5-3.6)	420	11.0 (10.0-12.2)
≥3[c]	197	17.3 (14.9-20.0)	46	4.1 (3.0-5.5)	243	21.3 (18.7-24.3)
Age at risk: 55-74						
Never-drinker	30	11.0 (7.7-15.9)	76	25.6 (20.4-32.2)	106	36.7 (30.2-44.5)
Ex-drinker[b]	34	14.5 (10.3-20.3)	68	26.2 (20.6-33.3)	102	40.6 (33.4-49.4)
<1[c]	595	14.0 (12.8-15.2)	874	19.9 (18.5-21.3)	1469	33.8 (32.1-35.7)
1 to <3[c]	230	17.7 (15.5-20.1)	258	21.0 (18.6-23.7)	488	38.7 (35.4-42.3)
≥3[c]	115	26.3 (21.8-31.8)	100	24.8 (20.3-30.3)	215	51.1 (44.5-58.7)
**Male ex-smokers (n=6821)**						
Age at risk: 35-54						
Never-drinker	2	1.7 (0.4-6.7)	2	1.6 (0.4-6.6)	4	3.3 (1.2-8.8)
Ex-drinker[b]	2	1.3 (0.3-5.2)	1	0.6 (0.1-4.4)	3	1.9 (0.6-6.0)
<1[c]	38	3.1 (2.2-4.3)	23	1.9 (1.2-2.9)	61	5.0 (3.8-6.5)
1 to <3[c]	8	4.4 (2.2-8.9)	2	1.1 (0.3-4.3)	10	5.5 (3.0-10.3)
≥3[c]	6	16.0 (6.9-37.0)	1	2.6 (0.4-18.7)	7	18.6 (8.7-39.9)
Age at risk: 55-74						
Never-drinker	5	5.3 (2.2-12.7)	15	14.5 (8.7-24.3)	20	19.8 (12.7-30.8)
Ex-drinker[b]	13	11.5 (6.6-19.9)	19	16.5 (10.4-26.0)	32	28.0 (19.7-39.7)
<1[c]	88	9.1 (7.4-11.2)	158	15.5 (13.2-18.1)	246	24.6 (21.7-27.9)
1 to <3[c]	15	11.6 (7.0-19.2)	26	19.6 (13.3-28.9)	41	31.2 (22.9-42.4)
≥3[c]	7	24.0 (11.4-50.6)	3	10.0 (3.2-31.1)	10	34.0 (18.2-63.4)
**Male never smokers (n=15 129)**						
Age at risk: 35-54						
Never-drinker	15	2.4 (1.4-3.9)	18	3.4 (2.1-5.6)	33	5.8 (4.1-8.2)
Ex-drinker[b]	7	5.0 (2.4-10.6)	2	1.5 (0.4-6.1)	9	6.5 (3.4-12.6)
<1[c]	97	3.4 (2.8-4.2)	58	2.4 (1.8-3.1)	155	5.8 (4.9-6.8)
1 to <3[c]	19	5.1 (3.2-8.0)	7	2.5 (1.2-5.3)	26	7.6 (5.2-11.3)
≥3[c]	12	19.4 (10.9-34.6)	3	6.4 (2.0-20.1)	15	25.8 (15.4-43.2)
Age at risk: 55-74						
Never-drinker	26	5.1 (3.5-7.6)	60	14.2 (11.0-18.4)	86	19.3 (15.6-24.0)
Ex-drinker[b]	6	5.1 (2.2-11.4)	12	10.8 (6.1-19.3)	18	15.9 (9.9-25.4)
<1[c]	113	4.9 (4.1-6.0)	247	13.3 (11.7-15.2)	360	18.3 (16.4-20.3)
1 to <3[c]	35	12.9 (9.2-18.0)	32	16.0 (11.3-22.7)	67	28.9 (22.7-36.8)
≥3[c]	19	24.5 (15.5-38.9)	7	13.4 (6.3-28.2)	26	37.9 (25.7-56.0)
**Female smokers (n=12 278)**						
Age at risk: 35-54						
Never-drinker	16	3.7 (2.3-6.1)	15	3.1 (1.8-5.2)	31	6.8 (4.8-9.8)
Ex-drinker[b]	2	2.8 (0.7-11.1)	3	4.2 (1.3-13.1)	5	6.9 (2.9-16.8)
<¼[c]	74	3.5 (2.7-4.4)	51	2.2 (1.6-3.0)	125	5.7 (4.7-6.9)
¼ to <1[c]	18	4.2 (2.6-6.7)	13	2.9 (1.7-5.1)	31	7.1 (5.0-10.2)
≥1[c]	32	8.9 (6.1-12.8)	6	1.6 (0.7-3.6)	38	10.5 (7.5-14.6)
Age at risk: 55-74						
Never-drinker	8	8.3 (4.0-17.1)	18	13.1 (8.1-21.1)	26	21.4 (14.3-31.9)
Ex-drinker[b]	2	8.4 (2.1-34.1)	7	26.9 (12.5-57.8)	9	35.2 (18.1-68.8)
<¼[c]	28	7.7 (5.2-11.3)	63	14.6 (11.2-18.9)	91	22.3 (17.9-27.6)
¼ to <1[c]	7	11.4 (5.3-24.5)	8	8.9 (4.4-17.9)	15	20.3 (12.1-34.0)
≥1[c]	24	32.9 (21.1-51.1)	11	12.0 (6.5-22.1)	35	44.8 (31.6-63.7)
**Female never smokers (n=57 481)**						
Age at risk: 35-54						
Never-drinker	23	0.6 (0.4-0.9)	68	1.4 (1.1-1.8)	91	2.0 (1.6-2.5)
Ex-drinker[b]	3	1.0 (0.3-3.0)	4	1.3 (0.5-3.4)	7	2.2 (1.0-4.7)
<¼[c]	134	1.0 (0.9-1.2)	147	1.0 (0.8-1.2)	281	2.0 (1.8-2.3)
¼ to <1[c]	7	1.0 (0.5-2.2)	6	0.8 (0.4-1.8)	13	1.8 (1.1-3.2)
≥1[c]	8	4.9 (2.4-9.8)	3	1.6 (0.5-5.0)	11	6.5 (3.6-11.8)
Age at risk: 55-74						
Never-drinker	130	2.4 (2.0-2.9)	433	8.0 (7.2-8.8)	563	10.4 (9.5-11.4)
Ex-drinker[b]	19	3.3 (2.1-5.2)	49	7.2 (5.4-9.6)	68	10.5 (8.2-13.4)
<¼[c]	185	2.1 (1.8-2.5)	537	6.8 (6.3-7.4)	722	9.0 (8.3-9.6)
¼ to <1[c]	25	7.6 (5.1-11.4)	21	6.8 (4.4-10.4)	46	14.4 (10.7-19.3)
≥1[c]	23	20.0 (13.2-30.2)	15	15.6 (9.4-26.0)	38	35.6 (25.8-49.0)

[a] Excludes people with no follow-up at ages 35-74 years or with evidence of pre-existing disease (self-reported cancer, MI, angina, heart failure, rheumatic heart disease, stroke, diabetes, TB, liver cirrhosis or chronic hepatitis) or quit drinking or smoking due to illness.

[b] Did not quit because of illness.

[c] Half-litre bottles of vodka per week.

Russia: 2-page baseline questionnaire (for phase 1 of recruitment, Jan-Dec 1999)

☐ City (1=Barnaul, 4=Tomsk)
☐☐ Interviewer number
☐☐☐☐ Household number (consecutive)
☐☐☐☐ Questionnaire number

Address..

Telephone.................................... Full name..

If proxy interview, give name of informant ....

☐ & proxy's relation to subject (0=self, 1=spouse, 2=sibling, 3=parent, 4=adult child, 5=other)

☐ Gender (1=male, 2=female) ☐☐ Age ☐☐ ☐☐ ☐☐☐☐ Date of birth (dd/mm/yyyy)

☐ Education completed (0=elementary, 1=secondary, 2=special secondary, 3=higher)

☐ Marital status (0=never married, 1=divorced, 2=widow(er)ed, 3=currently married)

☐ Profession (0=uniformed services, 1=labourer, 2=office worker, 3=self-employed, 4=housewife, 5=disabled, 6=unemployed, 7=retired, 8=other)

☐ Welfare (self-rating) (0=very poor, 1=poor, 2=fair, 4=good)

Medical history (questions 1-7)

Disease ever definitely or probably confirmed by a doctor (0=no, 1=yes), and, if so, approximate age when first diagnosed, and any important problems remaining (0=no, 1=yes)?

		Age at first onset	Still a problem?
1. ☐	Cancer? If Yes:		
☐☐	Site of origin (1=mouth or pharynx, 2=oesophagus, 3=stomach, 4=colon or rectum, 5=pancreas, 6=liver, 7=lung, 8=melanoma, 9=breast, 10=prostate, 11=bladder, 12=leukaemia or lymphoma,13=other, 0=unknown or multiple site)	☐☐	☐
2. ☐	Heart disease? If Yes:		
☐	Rheumatic heart disease	☐☐	☐
☐	Angina pectoris	☐☐	☐
☐	Myocardial infarction	☐☐	☐
☐	Heart failure	☐☐	☐
☐	Other heart disease (please specify..)	☐☐	☐
3. ☐	Stroke	☐☐	☐
4. ☐	Severe depression (impaired mood, indifference, feeling of hopelessness, etc)	☐☐	☐
5. ☐	Liver disease? If Yes:		
☐	Liver cirrhosis	☐☐	☐
☐	Chronic hepatitis	☐☐	☐
☐	Cholelithiasis	☐☐	☐
☐	Other liver disease (please specify..)	☐☐	☐

Russia: 2-page baseline questionnaire (for phase 1 of recruitment, Jan-Dec 1999)

	Age at first onset	Still a problem?
6. ☐ Any severe disability? If Yes:		
☐ Arthritis	☐☐	☐
☐ Deafness	☐☐	☐
☐ Blindness	☐☐	☐
☐ Memory loss	☐☐	☐
☐ Parkinson's disease	☐☐	☐
☐ Other	☐☐	☐

(please specify...)

7. ☐ Any medication on most days during the past year?
   - ☐ Anti-diabetic (specify)..
   - ☐ Anti-hypertensive..
   - ☐ Anti-inflammatory (specify, eg, aspirin)..
   - ☐ Other daily (specify medication and reason)...

Smoking

☐ Do you currently smoke? (0=no, 1=yes)

a) If No (ie, non- or ex-smoker):

☐ Ever smoked on most days for at least one year? (0=no, 1=yes)

☐☐ Years since stopped? ☐ Did you stop because you were ill (0/1)?

b) If Yes (ie, current smoker):

☐☐ How many cigarettes/day? (include "papirosa" cigarettes and hand-rolled cigarettes)

☐☐ Age began smoking

Alcohol

☐ Have you used any alcohol in the last year? (0=no, 1=yes)

a) If No (ie, non- or ex-drinker):

☐ Have you ever used alcohol at least once a month for a year?

☐☐ Years since stopped? ☐ Did you stop because you were ill? (0=no, 1=yes)

b) If Yes, then over the past year:

☐ How many days per week do you have some alcohol? (0=considerably less than weekly)

☐ How many days per week do you have some alcohol before noon?

How much vodka or other strong drink (about 40% or stronger) do you usually drink per week?
(0=none at all; if a range is given [eg, 2 or 3 bottles] then enter the upper end of the range)

☐☐☐☐ g per week or ☐☐ bottles per week (1 bottle = 500g = 0.5L)

What has been the most vodka you ever drank in one day?

☐☐☐☐ g per day or ☐☐ bottles per day

Name of interviewer (PRINT)...

Date of interview...

☐ Quality of interview: (0=poor, 1=fair, 2=good)

Russia: 5-page baseline questionnaire (for phase 2 of recruitment, 2002-2008)

ID barcode: ☐

Part 1: Identifiers

☐ City (1=Barnaul, 2=Byisk, 4=Tomsk)
☐☐☐☐☐ Household no. (allocated consecutively)
☐☐ Subject no. in household (allocated consecutively)
☐ Relation to decedent (1=relative, 2=neighbour, 3=non-relative at this address, 4=other of age 30-74)

Address...

Telephone...................................... Full name..

☐ Sex (1=M, 2=F) ☐☐ Ethnicity ☐☐ Age (30-74) ☐☐/☐☐/☐☐☐☐ Date of birth (dd/mm/year)

☐ Highest education completed (0=none, 1=primary, 2=secondary, 3=special secondary, 4=higher)
☐ Marital status (0=never married, 1=divorced, 2=widow(er)ed, 3=currently married)
☐ Present circumstances (0=very bad, 1=bad, 2=middle, 3=good)
☐ Have your circumstances changed in recent years? (0=worse, 1=no change, 2=improved)

Part 2: Education and employment

☐ Employed now (0=no, 1=yes)?

If Yes: ☐ Are you working at your specialty? (0=no, 1=yes)
☐ Have your job circumstances changed? (0=worse, 1=no change, 2=improved)
☐☐ Months unpaid in past year

If No: ☐ Why not? (0=retired, 1=lost job, 2=student, 3=invalid, 4=housewife)
☐☐ How many months have you been unemployed?

☐ Usual job (1=worker, 2=farmer, 3=salaried, 4=uniformed, 5=service sector, 6=sports, 7=none)
☐ Main work exposure (0=none, 1=radiation, 2=solvents, 3=oil, 4=asbestos, 5=pesticides, 6=welding, 7=dioxin)
☐☐ Industry (1=agriculture/forestry, 2=mining, 3=food/drink/tobacco, 4=textiles, 5=leather/fur, 6=wood/furniture, 7=paper/printing, 8=coke/oil, 9=chemicals/medicines, 10=rubber/plastics, 11=building materials, 12=metal production, 13=machinery, 14=electrical appliances, 15=vehicle manufacture, 16=utilities, 17=construction, 18=driver, 19=other)

Russia: 5-page baseline questionnaire (for phase 2 of recruitment, 2002-2008)

Part 3: Medical history:

Disease ever definitely or probably confirmed by a doctor (0=no, 1=yes), and, if so, approximate age when first diagnosed, and any important problems still remaining (0=no, 1=yes)?

		Age at first onset	Still a problem?
☐	Rheumatic heart disease	☐☐	☐
☐	Angina pectoris	☐☐	☐
☐	Myocardial infarction	☐☐	☐
☐	Heart failure	☐☐	☐
☐	Stroke	☐☐	☐
☐	Hypertension	☐☐	☐
☐	COPD	☐☐	☐
☐	Neoplasm (cancer); If Yes:		
☐☐	Site of origin of cancer (1=mouth or pharynx, 2=oesophagus,3=stomach, 4=colon or rectum, 5=pancreas, 6=liver, 7=larynx, 8=lung, 9=melanoma, 10=breast, 11=cervix, 12=other parts of uterus, 13=prostate, 14=bladder, 15=kidney, 16=thyroid, 17=leukaemia or lymphoma, 18=other, unknown or more than one site)	☐☐	☐
☐	Tuberculosis	☐☐	☐
☐	Liver cirrhosis	☐☐	☐
☐	Chronic hepatitis	☐☐	☐
☐	Chole-cystitis/-lithiasis	☐☐	☐
☐	Gastric ulcer	☐☐	☐
☐	Colorectal polyps	☐☐	☐
☐	Diabetes mellitus	☐☐	☐
☐	Severe depression	☐☐	☐
☐	Disability	☐☐	☐

☐ Any medication on most days during the last year (0=no, 1=yes)? If Yes:

- ☐ Anti-diabetic
- ☐ Antihypertensive
- ☐ Anti-inflammatory (eg, aspirin)
- ☐ Other daily medication

Russia: 5-page baseline questionnaire (for phase 2 of recruitment, 2002-2008)

Part 4: Tobacco

☐ Do you smoke? (0=no, 1=yes)

If Yes (ie, current smoker):

☐☐ How many cigarettes per day? (include "papirosa" and hand-rolled cigarettes; exclude pipe)
☐☐ Age began smoking (ie, using any type of tobacco on most days)
☐☐ How many years have you smoked?

If No (ie, never- or ex-smoker):

☐ Ever smoked (on most days for a year)? (0=no, 1=yes)

If yes:

☐ Age first began smoking
☐ Age last stopped smoking
☐ Years since you last stopped
☐ Did you stop because you were ill? (0=no, 1=yes)

If never-smoker:

☐ Did you ever live or work with a smoker? (0=no, 1=yes)

If Yes: Please enter the ages at which you were exposed to their smoke:

	From age	*To age*
Spouse(s)	☐☐	☐☐
Parent(s)	☐☐	☐☐
Colleague(s)	☐☐	☐☐

Part 5: Alcohol

☐ Do you drink any alcohol? (0=no, 1=yes)

If Yes, then:

☐ How many days/week do you have some alcohol (0-7)? (0 means none for most weeks)
☐ How many days/week do you have some alcohol before noon (0-7)? (0 means none for most weeks)

How much alcohol do you usually drink per week? (0=none at all; if a range is given, eg, 2 or 3 bottles, then enter the upper end of that range)

Vodka or other strong drinks (about 40% alcohol, or stronger)
☐☐☐☐ ml/week or ☐☐ bottles/week (vodka bottle=500ml)

Wine 20%
☐☐☐☐ ml/week or ☐☐ bottles/week (wine bottle=750ml)

Wine 10-12%
☐☐☐☐ ml/week or ☐☐ bottles/week (wine bottle=750ml)

Beer ☐☐ bottles/week (beer bottle=500ml)

Russia: 5-page baseline questionnaire (for phase 2 of recruitment, 2002-2008)

What was the most vodka you ever drank in one day?

☐☐☐☐ ml/day or ☐☐ bottles/day (vodka bottle=500ml)

☐ Have you ever drunk alcoholic liquids not intended for consumption (non-beverage alcohol) at least once a month for a year? (0=no, 1=yes)

[then go to Part 6]

If No (ie, non-drinker):

☐ Have you ever used alcohol at least once a month for a year? If Yes (used):

- ☐ How many days/week did you have some alcohol (0-7)? (0 means none in most weeks)
- ☐ How many days/week did you have some alcohol before noon (0-7)? (0 means none in most weeks)

How much alcohol did you usually drink/week?
If a range is given (eg, 2 or 3 bottles) then enter the upper end of the range (0=not at all)

Vodka or other strong drinks (40%)

☐☐☐☐ ml/week or ☐☐ bottles/week (bottle=500ml)

Wine 20%

☐☐☐☐ ml/week or ☐☐ bottles/week (bottle=750ml)

Wine 10-12%

☐☐☐☐ ml/week or ☐☐ bottles/week (bottle=750ml

Beer ☐☐ bottles/week (bottle=500ml)

What was the most vodka you ever drank in one day?

☐☐☐☐ ml/day or ☐☐ bottles/day (vodka bottle=500ml)

☐ Have you ever drunk spirit containing liquids not intended for consumption (non-beverage alcohol) at least once a month for a year? (0=No, 1=Yes)

☐☐ Years since you last stopped drinking any alcohol ☐ Did you stop because you were ill (0/1)?

Part 6: Diet

How often do you eat the following fresh fruits and vegetables?
(0=never, 1=no more than once a month, 2=1 to 3 times a month, 3=once or twice a week, 4=most days but not every day, 5=every day)

☐ Apples or pears

☐ Oranges and other citrus fruits

☐ Bananas

☐ Berries (in season)

☐ Greens and green onions (in season)

☐ Carrots

☐ Tomatoes (in season)

Russia: 5-page baseline questionnaire (for phase 2 of recruitment, 2002-2008)

Part 7: Blood pressure, height, weight and build (blank for absent subjects)

Blood pressure should be measured seated:

☐☐☐ / ☐☐☐ SBP / DBP before the interview

☐☐☐ / ☐☐☐ SBP / DBP after the interview

Height and weight should be measured without shoes in light clothing

☐☐☐ Height cm

☐☐☐ Weight kg

☐☐☐ Waist cm

☐☐☐ Hip cm

Part 8: Biological sample collection

If participant agrees, cut a sample of hair from back of head and seal in a bag with the ID number

☐ Hair sample provided? (0=no, 1=yes)

If subject agrees, take a sample of blood via capillary puncture of the finger (10 blood spots on special filter paper) and seal it in a bag with the ID number

☐ Blood sample provided (0=no, 1=yes)

Part 9: Description of interview

Name of interviewer (PRINT): ....

☐☐ Interviewer's identification number

☐☐/☐☐/☐☐☐☐ Date of interview (dd/mm/year)

☐ Quality of co-operation (0=poor, 1=fair, 2=good)
